# Role of Chromodomain-Helicase-DNA-Binding Protein 4 (CHD4) in Breast Cancer

**DOI:** 10.3389/fonc.2021.633233

**Published:** 2021-04-26

**Authors:** Apolonia Novillo, Ana Fernández-Santander, Maria Gaibar, Miguel Galán, Alicia Romero-Lorca, Fadoua El Abdellaoui-Soussi, Pablo Gómez-del Arco

**Affiliations:** ^1^ Department of Pre-clinical Dentistry, Faculty of Biomedical and Health Sciences, Universidad Europea de Madrid, Villaviciosa de Odón, Madrid, Spain; ^2^ Department of Medicine, Faculty of Biomedical and Health Sciences, Universidad Europea de Madrid, Villaviciosa de Odón, Madrid, Spain; ^3^ Department of Health Sciences, Faculty of Biomedical and Health Sciences, Universidad Europea de Madrid, Villaviciosa de Odón, Madrid, Spain; ^4^ Institute of Rare Diseases Research, Instituto de Salud Carlos III (ISCIII), Madrid, Spain

**Keywords:** breast cancer, *CHD4* gene, mutation, therapies in breast cancer, chromatin remodeling

## Abstract

Chromodomain-helicase-DNA-binding protein 4 (CHD4) is an epigenetic regulator identified as an oncogenic element that may provide a novel therapeutic target for the treatment of breast cancer (BC). CHD4—the core component of the nucleosome remodeling and deacetylase (NuRD) complex—may be mutated in patients with this disease. However, information on *CHD4* mutants that might allow their use as biomarkers of therapeutic success and prognosis is lacking. The present work examines mutations in *CHD4* reported in patients with breast cancer and included in public databases and attempts to identify their roles in its development. The databases revealed 81 point mutations across different types of breast cancer (19 of which also appeared in endometrial, intestinal, nervous system, kidney, and lymphoid organ cancers). 71.6% of the detected mutations were missense mutations, 13.6% were silent, and 6.2% nonsense. Over 50% affected conserved residues of the ATPase motor (ATPase and helicase domains), and domains of unknown function in the C-terminal region. Thirty one mutations were classified in the databases as either ‘deleterious’, ‘probably/possibly damaging’ or as ‘high/medium pathogenic’; another five nonsense and one splice-site variant were predicted to produce potentially harmful truncated proteins. Eight of the 81 mutations were categorized as putative driver mutations and have been found in other cancer types. Some mutations seem to influence ATPase and DNA translocation activities (R1162W), while others may alter protein stability (R877Q/H, R975H) or disrupt DNA binding and protein activity (R572*, X34_splice) suggesting CHD4 function may be affected. *In vivo* tumorigenecity studies in endometrial cancer have revealed R975H and R1162W as mutations that lead to CHD4 loss-of-function. Our study provides insight into the molecular mechanism whereby CHD4, and some of its mutants could play a role in breast cancer and suggest important implications for the biological comprehension and prognosis of breast cancer, identifying *CHD4* as a novel therapeutic target for BC patients.

## Introduction

Breast cancer (BC) is the most common type of cancer worldwide. At least five clinical subtypes have been identified at the molecular level: hormone receptor positive (progesterone receptor and/or estrogen receptor-positive or negative-HR+/−, *i.e.*, luminal A and luminal B), human epidermal growth factor receptor-2 positive (HER2-positive or ERBB2+), basal-like, normal-like, and triple-negative breast cancer (TNBC) (1–3). Hormone receptor positive/HER2 negative (HR+/HER2−) tumors account for 70% of all BC, HER2 positive tumors (HER2+) account for 15-20%, and triple-negative for 15% (4). This classification system helps oncologists prescribe the most appropriate treatment, *i.e.*, endocrine therapy, chemotherapy (alone or combined), and/or HER2-targeted therapy (2, 5, 6). Being able to predict tumor behavior avoids over-treating patients likely to respond well, while those less likely to do so can be given more aggressive treatment (7–10).

Epigenetic factors that mediate reversible changes at the chromatin level may be involved in regulating tumorigenesis, as well as the plasticity and heterogeneity of tumor cells in BC (11–13). Identifying these factors and the signaling pathways they mediate could help reveal candidates for next-generation anti-cancer drugs. One such epigenetic regulator, chromodomain-helicase-DNA-binding protein 4 (CHD4)—a chromatin remodeler that can reposition, eject and replace histones within the nucleosome using energy from the hydrolysis of ATP—may possess oncogenic and treatment resistance-related activities in different cell types. The ATPase subunit of CHD4 (also known as Mi-2*β*) is the major catalytic component of the nucleosome remodeling and deacetylase (NuRD) complex, also including the histone deacetylases HDAC1 and 2, among other proteins. CHD4/NuRD therefore regulates chromatin accessibility, transcription, chromatin assembly, the response to DNA damage, the maintenance of genome integrity, and progression through the cell cycle; it has also been associated with the formation of metastasis (14–17). In addition, CHD4/NuRD is reported to be involved in lineage commitment in the immune system, the nervous system, and in striated muscle differentiation (18–20). CHD4 has also been implicated in the regulation of transcriptional events involved in oncogenesis and cancer progression through different molecular pathways in several types of cancer. Certainly, it has been implicated in the maintenance of cell stemness in a hepatocellular carcinoma model (21), and its overexpression is associated with poor prognosis in several cancer types, including BC (21–28). Indeed, in the PANCAN study, *CHD4* was identified as one of the 12 most important cancer-driving genes involved in chromatin epigenetics (29, 30). Moreover, the large number of somatic mutations in *CHD4* seen in different cancer types (carcinomas, gliomas, medulloblastoma, hematopoietic, and lymphoid) in different tissues (gynecological, nervous system, lymphoid organs, intestine, kidney, lung, *etc.*) (31–38) make this epigenetic regulator worthy of attention.

The present work collates *CHD4* mutations in patients with BC as recorded in public databases and attempts to identify their roles in this disease. The literature regarding *CHD4* as a prognostic biomarker and potential therapeutic target in BC is also discussed.

## Material and Methods

Three databases—the cBioPortal (39), COSMIC (40), and TCGA-BRACA (https://www.cancer.gov/tcga) databases—were searched to identify somatic mutations in *CHD4* in patients with BC. These databases provide information on *CHD4* mutations in different types of cancer, as well as their potential functional impact (as determined by the Mutation Assessor, Polyphen-2 and SIFT tools). The following keywords were used in searches: somatic mutations (confirmed and/or previously reported), tumor sample, mutant impact (pathogenic and neutral), and mutation type (single mutation, small insertions and deletions, frameshifts and splice variants). Copy number variations and fusions were not taken into account. Additional information, regarding *CHD4* as a potential therapeutic target was sought *via* a PubMed search (articles written in English; keywords: breast cancer, cancer risk, CHD4, *CHD4* mutations, and CHD4 breast cancer).

## Results

### Somatic *CHD4* Mutations in Breast Cancer

**Figure 1:**
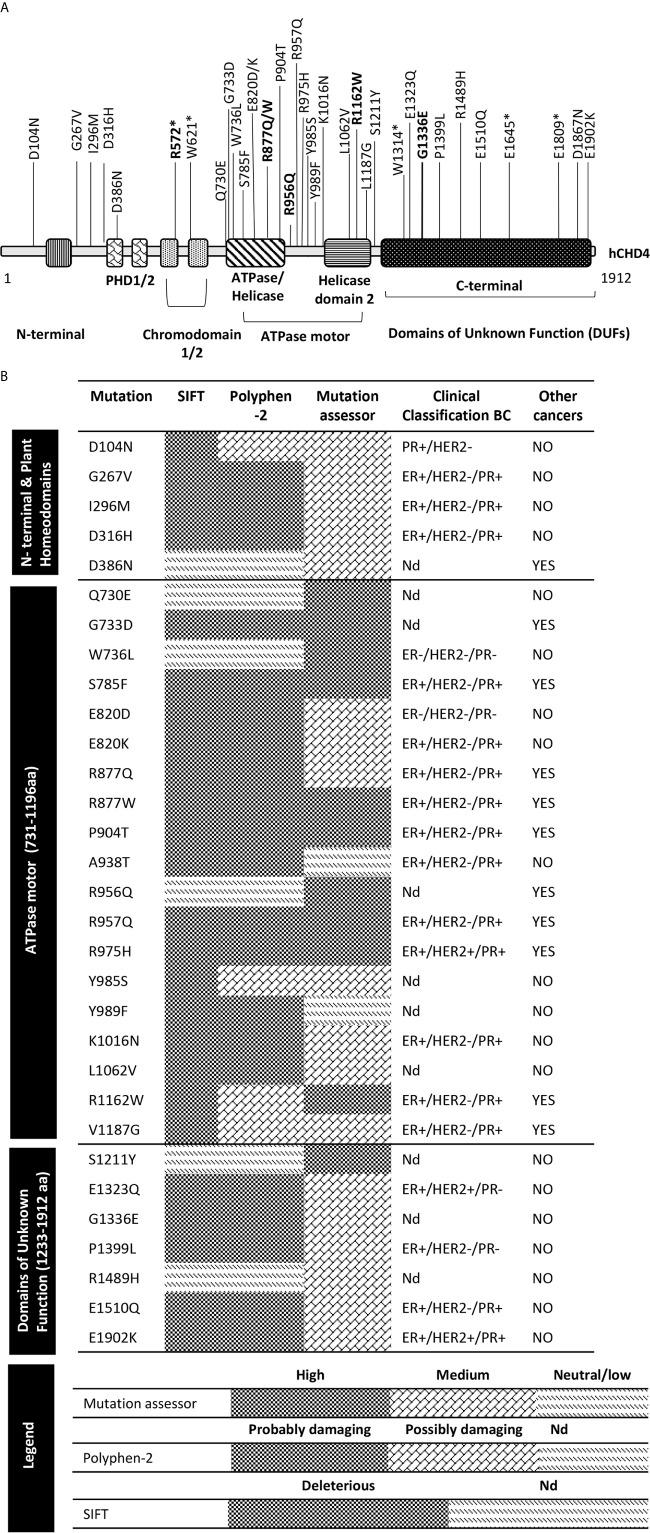
Overview of CHD4 protein structure and somatic mutations in BC tumors analyzed in this study. **(A)** Schematic structural representation of CHD4 protein, showing its domains and the locations of the potential damaging point mutations found in BC patients. N-terminal domain, was first characterized through nuclear magnetic resonance (NMR) spectroscopy analysis. This domain is implicated in directing CHD4 to DNA double strand breaks, thus enhancing its chromatin remodeling properties (41). The Plant homeodomains (PHD1/2) are characterized by conserved Cys-His-Cys modules (42). Each PHD has the ability to specifically bind to the amino-terminal tails of histone 3 (H3) of the nucleosomes in an independent and simultaneous way (43). Chromodomains (CHD1/2), are highly conserved domains found in a large array of organisms, from protozoans to mammals. These domains display DNA and nucleosome binding activities (44–45). The ATPase motor, is common to all CHD protein family members and has important roles in nuclear processes, such as transcriptional regulation, chromosomal maintenance and stability, and it is critical for the ATPase/helicase function. Mutations in this domain often lead to severe consequences in patients with developmental syndromes such as Sifrim–Hitz–Weiss (46–47) and in cancer (32, 48–49). Regarding the Domains of unknown functions (DUFs), CHD4 contains two of these domains (DUF1/2) in the C-terminal part of the protein (50). This domain may possess a repressive transcriptional activity, since CHD4 and CHD3 have been described to interact through this region with numerous co-repressors (hunchback, NAB2, RFP among others). **(B)** Somatic *CHD4* mutations in BC patients discussed in this work: (i) Prediction of the functional effect of the *CHD4* mutations in the protein activity, according to the following criteria: deleterious (according with SIFT) and probably/possibly damaging (according to polyphen), and/or high/medium (according to mutation assessor); (ii) presence of these mutations in different BC clinical types; (iii) presence of these mutations in other cancers. PR+, progesterone receptor positive; ER+, estrogen receptor positive; PR−, progesterone receptor negative; ER−, estrogen receptor negative; HER2+, human epidermal growth factor receptor-2 positive; HER2−, human epidermal growth factor receptor-negative; Nd, non-available data; hCHD4-human chromodomain-helicase-DNA-binding protein 4. *stop codon.

The database search revealed that 253 BC patients (3%) had somatic mutations, fusion and variation in copy number in the *CHD4* gene. In this study, we focus only on the 81 point mutations found in these tumors (**Supplementary Table 1**). The majority were found in all the checked databases, although a small percentage (<1%) was recorded only in the cBioPortal database. The *CHD4* point mutations were found in several types of BC (lobular and ductal carcinoma, invasive, metastatic, neuroendocrine, phyllodes), with different BC clinical classification [see **Supplementary Table 1** and **Figure 1**, (41–54)]. Nineteen of them were also found in endometrial, intestinal, nervous system, kidney, and lymphoid tissue cancers (**Supplementary Table 1**). These 81 mutations, classified as either single nucleotide polymorphisms (SNPs), small insertions/deletions, frameshifts or splice variants, occurred in different places across the whole gene. Under 5% were small deletions or insertions; <3% involved splicing sites (**Table 1**). Most of these 81 mutations were missense (71.6%), followed by coding-silent (13.6%) and nonsense (6.2%). Mutations influencing BC phenotypes were observed to affect CHD4 PHD finger 2, the double chromodomain (CHD), both lobes of the ATPase motor (ATPase and helicase domains), and the C-terminal domains of unknown function (DUFs) (**Figure 1**). Over 50% of these mutations affected the ATPase motor and the C-terminal DUFs (**Supplementary Table 1** and **Figure 1**).

**Table 1:** *CHD4* mutations in breast cancer.

Type	Total number	%	Potential pathogenic mutations and/or putative driver mutations
Missense-Type SNP	58	71.6	D104N, G267V, I296M, D316H, D386N, Q730E, G733D, W736L, S785F, E820D, E820K, **R877Q**, **R877W**, P904T, R956Q, R957Q, **R975H,** Y985S, Y989F, K1016N, I1062V, **R1162V,** L1187G, S1211Y, E1323Q, G1336E, P1399L, R1489H, E15102, D1867N, E1902K,
Nonsense	5	6.2	R572*, W621*, W1314*, E1645*, E1809*
Coding silent	11	13.6	
Deletion	4	4.9	**K119del, E139del**
Splice site	2	2.5	**X34_splice**
Insertion	1	1.2	
Total	81	100	

In bold are highlighted the putative driver mutations in CHD4. *stop codon.

Six point mutations detected in BC samples—R572*, R877Q, R877W, P904T, R975H and R1162W—were also frequently detected in endometrial cancers (**Supplementary Table 1**). R877Q/W and R1162W are located in the ATPase and helicase 2 domain, respectively (**Figure 1**), involving residues highly conserved both evolutionarily speaking and among closely related gene family members.

### Prediction of the Functional Effects of *CHD4* Mutations in Breast Cancer

**Supplementary Table 1** shows the data extracted from the different databases for the 81 mutations detected, and their classification as either deleterious according to the SIFT tool, probably/possibly damaging according to the Polyphen tool, and/or as high/medium pathogenic according to the Mutation Assessor tool (see **Figure 1**). According to these classifications a total of 31 mutations were identified as pathogenic, suggesting that these mutations may disrupt the function of the CHD4 protein. (Note: It should be remembered that few functional studies have been undertaken to confirm these predictions). Nineteen of these 31 mutations are located in the ATPase motor subunit. Five further nonsense mutations (R572*, W621*, W1314*, E1645*, and E1809*) not classified by the above tools but known to produce truncated proteins, were also deemed likely harmful. R572* is of particular interest because it has been reported in different cancers of the endometrium and large intestine. However, the bioinformatics tools used did not allow for classification of the following mutations as pathogenic or not: a) the single splice mutation X34_splice, and b) two small in-frame deletions K119del and E139del. These mutations are located in the N-terminal region of CHD4 possibly giving rise to alternative proteins that could also be harmful (see next section).

### Putative Driver Mutations of *CHD4* in Breast Cancer

A further search of cBioPortal served to identify eight putative driver mutations [see **Table 2**, (39, 55–57)]. One of these is a splicing type mutation located at residue 34 detected in invasive lobular carcinomas and uterine endometrioid carcinoma. This variant is associated with exon 1 splicing donor disruption (G>A) that could lead to a short truncated protein of 51 aminoacids (transcript ID ENST00000644132.1; protein ID A0A2R8Y539), or to nonsense-mediated decay. Therefore, it is predicted that the mutation impact of X34_splice is loss of CHD4 function. The other two in-frame small deletions K119del and E139del, have been identified in ductal, invasive ductal, and mixed carcinomas; and in other tissues such as central nervous system (see **Table 2**). No information about possible impacts on protein functionality is available for these mutations. Hence, it could be that these mutations could affect transcription leading to nonsense-mediated decay or to the synthesis of a CHD4 protein with reduced stability or activity.

**Table 2:** Putative driver mutations in the *CHD4* gene according to cBioPortal.

Mutation	Breast cancer tumor type	Other tumors	Mutation impact	Location in the protein	Predicted effect based on available information	Reference
X34_splice	ILC	Uterine endometrioid carcinoma	Loss of function	N-terminal	Truncated protein or NMD No DNA binding, No functional remodeling, No ATPase activity	(39) This study
K119del	MDL, IDC	Large intestine, prostate, central nervous system	No information	N-terminal	Harmful protein or NMD	(39)
E139del	IDC	Soft tissue, salivary gland, hematopoietic, lymphoid tissue	No information	N-terminal	Harmful protein or NMD	(39)
R572*	IDC	Endometrium, large intestine	Loss of function	CHD1	Truncated protein Reduced DNA binding	(39, 55)
R877W	ILC, IDC, BRACA	Endometrium, stomach, hematopoietic, lymphoid tissue	Pathogenic SIFT: 0 Polyphen-2:0.99	ATPase motor	Disruption of protein function	(39)
R877Q	IDC	Endometrium, large intestine, hematopoietic, lymphoid tissue	Pathogenic SIFT: 0 Polyphen-2: 1	ATPase motor	Disruption of protein function	(39)
R975H	IDC	Endometrium, large intestine, pancreas, kidney, hematopoietic, lymphoid tissue	Loss of function Pathogenic SIFT: 0 Polyphen-2:0.99	ATPase motor	Reduced protein stability Disruption protein function	(39, 56)
R1162W	IDC	Endometrium, large intestine	Loss-of-function Pathogenic SIFT: 0 Polyphen-2: 0.84	ATPase motor	Reduced protein stability Disruption of interaction with ATP. Reduced ATPase activity	(39, 55–57)

ILC, invasive lobular carcinoma; IDC, invasive ductal carcinoma; MDL, mixed ductal and lobular carcinoma; BRACA, breast invasive carcinoma; NMD, nonsense mediated decay, CHD1, Chromodomain 1. In polyphen-2 reporter, the scaled score ranges between 0 and 1, in which scores closer to 1 indicate that amino acid substitution is damaging, and scores closer to 0 indicate that it is neutral. In SIFT prediction, scores range from 0.0 (deleterious) to 1.0 (tolerated). The score can be interpreted as follows: 0.0 to 0.05—variants with scores in this range are considered deleterious. *stop codon.

A further four point mutations (R8777Q/W, R975H, R1162W) are located in the ATPase motor domain. This site plays important roles in chromatin remodeling with impacts on transcriptional regulation, chromosome maintenance and stability, and is therefore critical for ATPase/helicase function. These missense mutations are found in invasive ductal carcinomas and have been identified in other tissues such as endometrium, intestine, and hematopoietic and lymphoid tissues (see **Table 2**). The last putative driver mutation examined here is a nonsense one (R572*) located in the first chromodomain (CHD1), with a role in DNA and nucleosome binding activities. It is found mainly in invasive ductal cancer but has also been observed in other tissues such as endometrium and large intestine.

## Discussion

### Somatic Mutations of *CHD4* in Breast Cancer

Many somatic mutations of *CHD4* have been reported to be associated with different carcinomas, gliomas, medulloblastoma, hematopoietic, and lymphoid carcinomas (31–36, 47). In the last decade, large-scale exome sequencing has revealed a *CHD4* gene mutation frequency of 17–20% in different types of endometrial cancers (32, 36), and the gene is also frequently mutated in different types of gynecological cancers (37, 38).

The present results confirm that *CHD4* is also mutated in certain types of BC, but at a lower frequency than in other gynecological cancer (<3% of the BC tumors examined showed somatic mutations in *CHD4*). Some of these mutations may change or promote the loss of CHD4 activity, in some cases driving oncogenic transformation (17, 32, 56) and perhaps leading to specific cancerous phenotypes (driver mutations). Other mutations are considered passenger mutations that confer no growth characteristics but that just happened to be present in the ancestor cancer cell line when it acquired one of its driver mutations (58).

The present work reports on 81 mutations in the *CHD4* gene (**Supplementary Table 1**). Using bioinformatics tools, we identified 36 as potentially harmful (**Figure 1**) and eight of them as putative drivers of cancer (**Table 2**). Nineteen of the 36 potentially harmful mutations located in the ATPase/helicase functional domain of CHD4 are highly conserved residues and have been also found in endometrial cancer (32, 56, 59). The eight *CHD4* mutations identified here classified as putative drivers mutations (see **Table 2**) could confer a growth advantage to cells carrying them and have been positively selected during the evolution of breast cancer. These driver point mutations observed in breast cancer patients are located in the first chromodomain (R572*) and mainly in the ATPase motor domain (R877Q/W; R975H, R1162W). According to their location, it is clear that the majority of these putative *CHD4* driver mutations in breast cancers negatively affect CHD4-ATPase and/or its remodeling activity (**Table 2**). It is conceivable that a reduction in overall CHD4 activity in healthy cells contributes to cancer genesis and progression. Further, although less frequent, other identified *CHD4* gene frameshift small deletions or splicing mutations (X_34splicing; K119del, and E139del) will similarly affect CHD4 activity.

Effects on the activity of some *CHD4* mutations have been examined (i) using *Drosophila melanogaster* CHD4 homolog dMi-2 as a model (55), (ii) by cryoelectron microscopy to study the structure of *Homo sapiens* CHD4 engaged with a nucleosome core particle (57), (iii) in functional studies of engineered cancer cells (56), and (iv) using bioinformatics functional annotation tools (SIFT, Polyphen-2, Mutation Assessor). Some *CHD4* mutations influence CHD4 ATPase and DNA translocation activity (R1162W, H1196Y, H115R, and L1215P), while others seem to alter the protein’s stability (L912V and C464Y) or disrupt its DNA binding activity (V558F and R572Q). For example, Residue 572 located in the CHD is involved in DNA-binding, and functional studies have shown SNP R572G to be involved in disruption of contact with the tracking DNA strand, reducing DNA binding affinity, thus provoking the loss of full remodeling and ATPase activities (55).

Few functional studies are available to confirm the harmful nature of the 31 somatic mutations predicted and/or the impacts of the eight putative driver mutations reported in this study (see **Figure 1** and **Table 2**). However, the effects of the hot-spot mutations R975H and R1162W have been investigated in endometrial cancer cells (56). These mutations showed no impairment of CHD4–DNA interaction or NuRD complex formation, but did show reduced CHD4 protein stability, mimicking a loss of function leading to the up-regulation of the cancer stem cell marker CD133. This phenotype was then verified by invasive capacity, spheroid formation, and *in vivo* tumorigenicity studies. Patients with mutant *CHD4* also showed overexpression of CD133. The authors concluded that these mutations can promote endometrial tumorigenesis through the TGF*β* signaling pathway, and that endometrial cancer might be treated by TGF-beta inhibition. In agreement with these observations, functional analyses indicate that the arginine at position 1162 in the ATPase motif VI forms an arginine finger that interacts with the ATP analog adenylyl imidodiphosphate (AMP-PNP) within the protein itself, and that replacing this residue with glutamine impairs ATP hydrolysis (55, 57), revealing how CHD4 function is perturbed at the molecular level.

The R572*, R877Q/W, P904T, R975H, and R1162W mutations detected in the present BC samples all involve highly conserved residues. These mutations have been identified in endometrial, large intestine, hematopoietic, and lymphoid cancers (**Supplementary Table 1**). In particular, R877Q/W and R1162W are located in the ATPase domain and in the helicase domain respectively (**Figure 1**), being strongly conserved residues. When these residues undergo germline or *de novo* mutation in *CHD4*, they cause Sifrim–Hitz–Weiss syndrome or neurodevelopmental disease. The same corresponding mutations occurring in *SMARCA1*, *SMARCA4*, and *SMARCA2* (components of a closely related chromatin remodeling complex known as SWI//SNF) cause Schimke immune-osseous dysplasia, Coffin–Siris syndrome and Nicolaides–Baraitser syndrome (32, 47, 53, 54). These findings strongly support the idea that the somatic mutations in the ATPase/helicase domain of CHD4 are associated with BC. The mechanisms whereby these variants may influence breast tumorigenesis are largely unknown and merit further investigation.

More functional studies are needed to classify *CHD4* mutations as driver or passenger mutations and to thus determine whether they contribute to breast cancer genesis. Our results show that missense mutations (76%) predominate, so we suggest that the mutated CHD4 protein itself may contribute to tumor transformation *via* gain of function or dominant negative mechanisms. Considering the functional data available for some *CHD4* mutations (see **Table 2**), it is possible that these mutations cause changes in the position of some nucleosomes *in vivo*, thus making regulatory sequences that control gene transcription more or less accessible. This could therefore be the molecular mechanism that contributes to activation of oncogenes or repression of tumor suppressor genes (17, 23), consistent with the control by CHD4 of repression of tumor suppressor genes during DNA damage (16, 38). As the ATPase function of CHD4 is required for this process, more work is needed to elucidate the functional consequences of mutations that lead to reduced ATPase catalytic activity (*e.g.* R877Q/W, R975H, and R1162W). *CHD4* mutations might also affect the whole assembly and activity of the NuRD complex. NuRD combines CHD4 nucleosome remodeling activity with HDAC1/2 histone deacetylase activities, and other proteins are part of this complex (MTAs, MBD2/3, GATAD2A/B, and RBBP4/7). Early work has suggested that remodeling is a pre-requisite for efficient nucleosome deacetylation. Thus, altered CHD4/NuRD assembly and complete activity are likely to have an impact, not only on DNA binding or nucleosome positioning, but also on histone acetylation.

Breast tumor initiation and progression are predominantly driven by acquired genetic alterations, although micro-environmental and epigenetic changes also play an important role in these processes (60). Our analysis suggests that *CHD4* mutations are found in different types of breast cancer, concurring alongside mutations in tumor suppressor genes or oncogenes including *BRCA2*, *TP53, ERBB2, PIK3CA, E2F3, ATM, etc.* (data not shown), as have been indicated by other authors’ (38). The individual contribution of *CHD4* mutations to malignant transformation is, therefore, difficult to assess due to the few functional data available. However, *CHD4* mutations found in BC tumors have revealed defects in different structural domains that could give rise to diverse effects such as lower ATPase activity, decreased nucleosome binding, inefficient coupling of ATPase and remodeling activities, and altered nucleosome positioning (*e.g.* R877Q/W, R975H, R1162W, R572*). This heterogeneity of defects and consequences implies that the presence of *CHD4* mutations in breast cancer cells may create different epigenetic frameworks that could affect the progression of breast cancer in interplay with mutations in other genes. The interpretation of all this information may benefit BC patients.

### CHD4 in Breast Cancer: Lessons From *In Vitro CHD4* Loss of Function Studies

**Figure 2:**
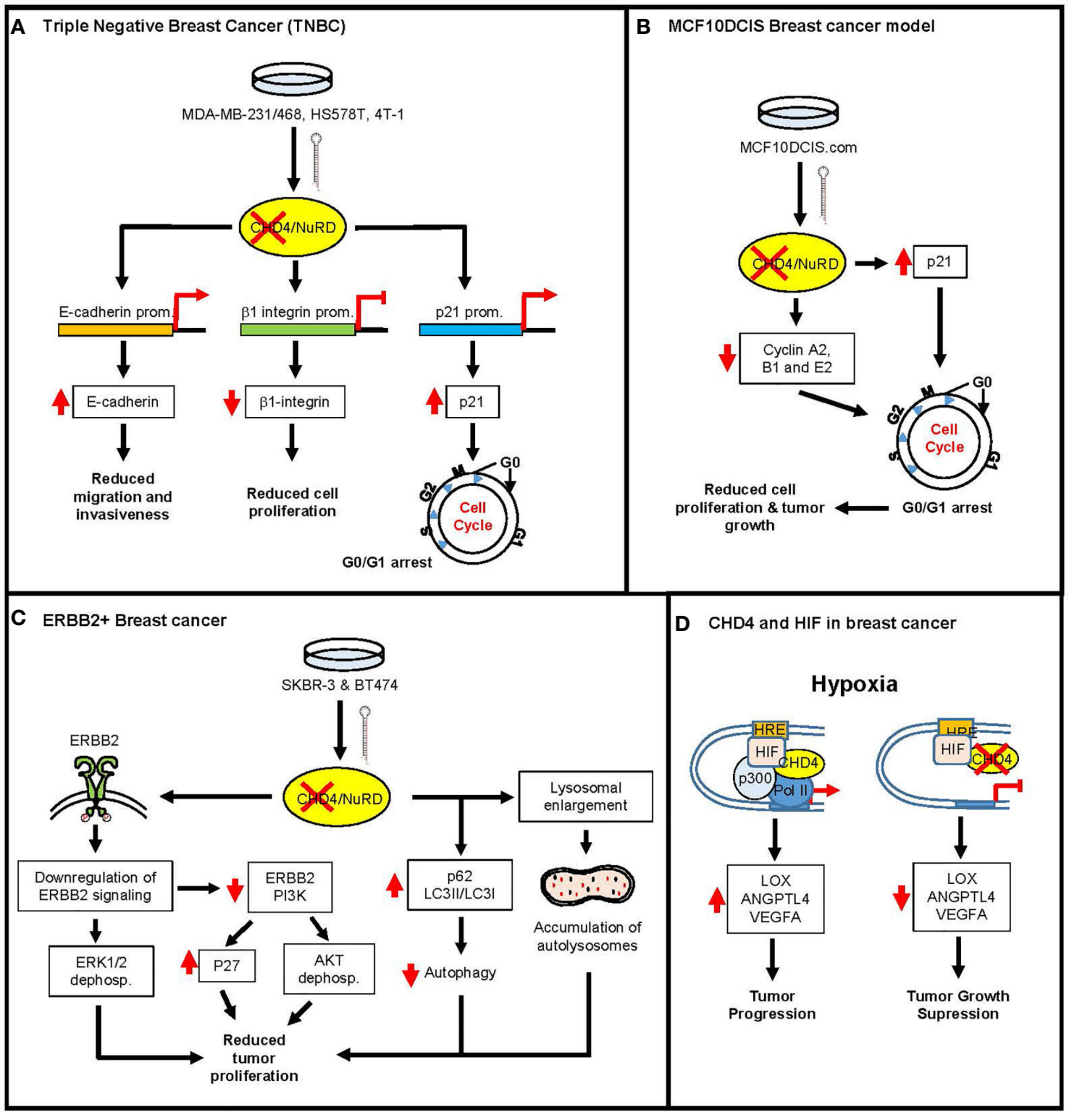
Cellular mechanisms of actions of *CHD4*-knock down in different BC models. **(A)** *CHD4*-KD in TNBC cells. Left; MDA-MB-231/468 and HS578T BC cells. Luo et al. (26) reported the *E-cadherin* transcriptional repression by HDAC/NuRD-dependent deacetylation of its promoter. Knockdown of *CHD4* induced hyperacetylation of *E-Cadherin* promoter, E-cadherin protein accumulation and the subsequent Reduction in migration and invasiveness. Middle; 4T-1 BC cells. Ou-Yang et al, 2019 (27) showed that CHD4 transcriptionally regulates β1-integrin expression to regulate BC cell proliferation. Furthermore, *CHD4*-KD in these cells produces β1-integrin downregulation and reduced cell proliferation. Right; MDA-MB-231 and HS578T BC cells. Hou et al., 2017 (25) described HDAC1 recruitment to *CDK1A* (p21) promoter and repression of p21. *CHD4*-KD in this case induced accumulation of p21 and G0/G1 arrest. **(B)** *CHD4*-KD in MCF10DCI.com BC cell line. D’Alesio et al. (61) reported the effect of the *CHD4* silencing in these cells. They found that the down-modulation of CHD4 induced a G0/G1 cell cycle arrest due to a p53-independent p21 accumulation and downregulation of Cyclins A2, B1, and E2, with the subsequent reduction in cell proliferation and mouse-xenograft tumor growth. **(C)** CHD4 in ERBB2+ BC cells. D’Alesio et al. (62) reported the effect of the *CHD4*-KD in two ERBB2+ BC cell lines (SKBR-3 and BT474). In these cells, CHD4 regulated both ERBB2 signaling and autophagy. *CHD4* silencing in these cells resulted in ERBB2 Tyr^1248^ phosphorylation, ERK1/2 and AKT dephosphorylation, and downregulation of both ERBB2 and PI3K protein levels. *CHD4* KD also late stages of autophagy, with increased levels of p62, LC3II/I ratio and lysosomal enlargement and an accumulation of autolysomes, everything resulting in reduced tumor proliferation. **(D)** CHD4 mediates HIF activation in BC during hypoxia. Upon hypoxia (28), CHD4 recruits HIF1/2a to the Hypoxia Response Elements (HRE), together with p300, thus stimulating the transcription of a subset of HIF target genes, such as LOX, ANGPTL4, and VEGFA, promoting tumor progression (left). *CHD4* silencing would inhibit this tumor growth (right).

The role of CHD4 in transcriptional gene regulation in both normal and cancer cells has been well documented. As a component of the NuRD complex, CHD4 controls chromatin accessibility and mediates the downregulation of many genes whose products contribute to the DNA damage response, tumor malignancy, and cell cycle control (16, 17, 22, 24, 29–31). An oncogenic role for *CHD4* has been defined in initiating and supporting tumor suppressor gene (TSG) silencing in human colorectal cancer (23). Further, there is evidence that, in response to DNA damage, CHD4 can act in a NuRD dependent or independent manner (17). In this context, *CHD4* mutations that diminish its function could increase the sensitivity to some treatments and therefore the interpretation of these mutations could benefit patients. Less attention has been paid to the role of *CHD4* in breast cancer, and the known implications of *CHD4* mutations in this pathological process are scarce. We here review *in vitro* data arising from loss of function studies in different types of breast cancer cells using *CHD4* knockdown models (**Figure 2**). These studies are relevant to understand the role of *CHD4* in different types of breast cancer, and help interpret *CHD4* mutations in breast cancer. There are different molecular mechanisms by which CHD4 interacts with different factors to promote cancer development according to the cell context (see **Figure 2**). In BC cells, several authors have described that: (i) through transcriptional mechanisms, CHD4 regulates downstream pathways essential for cellular proliferation, migration, invasiveness, differentiation, and autophagy (25–28, 61–64). Further, CHD4 has been identified as an oncogene involved in epigenetic suppression of multiple tumor suppressor genes *via* modulation of promoter activity (25–27) (**Figures 2A, B**); (ii) CHD4 maintains genome stability in BC cells through non-transcriptional mechanisms and cell proliferation (**Figure 2C**).

In triple negative breast cancer cells (TNBCs), *CHD4* regulates *β*1 integrin, E-cadherin, and p21 expression to control cell migration, invasiveness, and proliferation through different mechanisms (25–27) (**Figure 2A**). Data from *CHD4*-KD studies in human TNBC cells (MDA-MB-231, Hs578T, MDA-MB-468), have shown diminished *β*1 integrin expression at the level of protein and mRNA, suggesting that CHD4 works as an upstream mediator. Thus, an important role was described for CHD4 as a mediator in epithelial–mesenchymal transition (EMT), as vimentin, *ß*-catenin, and Snail expression were also reduced in these human TNBC cells (27). Using next-generation sequencing and bioinformatics techniques, these authors were able to confirm that CHD4 regulates *β*1 integrin in TNBC cells, indicating that the CHD4–*β*1 integrin axis might serve as a predictive marker of prognosis in patients with this disease (27). These *in vitro* findings were confirmed *in vivo* since co-expression of *β*1 integrin and *CHD4* was directly linked to the appearance of metastasis and disease recurrence in TNBC patients (27).

The role of CHD4 in the malignant behavior of TNBC (MDA-MB-231, Hs578T, MDA-MB-468) and non-TNBC (H184B5F5) cell lines was examined by Luo et al. (26). These authors reported that high levels of *CHD4* correlate positively with cell motility and mortality and that CHD4 mediates epithelial–mesenchymal transition (EMT) through E-cadherin, N-cadherin, and fibronectin expression. At the molecular level, *CHD4* silencing promotes the up-regulation of E-cadherin through the hyperacetylation of histone H3 at the E-cadherin promoter, reducing migration and invasiveness (**Figure 2A**).

It has also been reported that CHD4 deficiency impairs cell survival by increasing the expression of p21 in BRCA-proficient BC cell lines (MDA-MB-231, Hs578T cells), *i.e.*, models of TNBC (25) (**Figure 2A**). The authors indicate that CHD4 deficiency impairs the recruitment of histone deacetylase 1 (HDAC1) to the *p21* promoter, inducing its transcription, *CHD4* KD increased the sensitivity of these cells to cisplatin. The knockdown of p21 in these CHD4-depleted cells therefore overcomes cisplatin resistance and poly-(ADP-ribose) polymerase (PARP) inhibitor-mediated growth suppression (25).

In human TNBC (BT-549) cells, it was recently shown that the MUC1-C-dependent activation of CHD4, as part of the NURD complex, drives the differentiation of cells from luminal to basal (64). In this case, the oncogenic mucin 1 C-terminal subunit (MUC1-C) was reported to bind directly to the MYC HLH-LZ domain that, in turn, positively regulates the transcriptional expression of some NuRD complex components, such as MTA1 and MBD3. In contrast, MUC1-C/MYC does not affect *CHD4* mRNA levels but increases its protein expression at the post-transcriptional level in basal but not luminal BC cells.

In addition to transcriptional regulation, emerging data indicate that CHD4 also plays important roles in other processes that ensure proper DNA replication, cellular integrity, proliferation and genome integrity. Therefore, non-transcriptional mechanisms by which CHD4 maintains genome stability are also being described in BC cells (63). Using genome-wide shRNA screening in BRCA2 ovarian (PEO1) and BC cells (HCC1937, SUM1315MO2, both BRCA1 mutant), it was found that the loss of CHD4 alters the response to cisplatin (63). At the molecular level, the same authors demonstrated that CHD4 depletion enhances PCNA monoubiquitilation only in the ovary BRCA2 mutant cells. Further, CHD4 depletion was found to enhance *γ*H2AX foci and reduce the proliferation of BRCA1 BC cells (HCC1937, SUM1315MO2).

The construction of epigenetic libraries *via in vitro* and *in vivo* shRNA screening in human BC cells (MCF10DCIS.com line) recently identified CHD4 as an essential regulator of BC growth (61) (**Figure 2B**). Moreover, *in vitro* CHD4 depletion in TNBC (one of the most aggressive BC subtypes) was reported to significantly reduce cell proliferation and migration, and dramatically reduce tumor mass *in vivo* (61). The same was also seen in luminal B and triple negative PDX models, as well as in a transgenic mouse model (MMTV/NeuT), all of which expressed an activated rat ERBB2 ortholog (61). These authors suggested that the pharmacological inhibition of CHD4 might improve the treatment of TNBC and could also overcome resistance to approved drugs in the case of HER2+ breast cancers. In addition to its involvement in chromatin assembly, CHD4 seems to regulate BC cell cycle progression, and its silencing leads to the accumulation of cells in the G0 phase, a dramatic reduction of DNA synthesis, and an up-regulation of the tumor suppressor p21, independent of p53 (61). In fact, the depletion of CHD4 in the human mammary epithelial cell line MCF10A abrogated its tumorigenic potential without affecting cell proliferation and migration. This strongly suggests that CHD4 could be targeted to impair BC progression (61).

How CHD4 might promote the progression of BC is still a matter of debate. Work with HER2+ BC cell lines, SKBR-3 (ER-, PR-) and BT474 (ER+, PR+) has shown that CHD4 depletion leads to a significant inhibition of cell proliferation, inducing p27KIP1 up-regulation, Tyr1248 HER2 phosphorylation, ERK1/2 and AKT dephosphorylation, as well as the downregulation of both HER2 and PI3K (62) (**Figure 2C**). It is speculated that CHD4 depletion might have an inhibitory effect on the downstream ERBB2 signaling cascade, due to a post-translational mechanism. In addition, *CHD4* silencing was reported to impair the late stages of autophagy, resulting in increased levels of LC3 II and SQSTM1/p62 proteins, lysosomal enlargement and the accumulation of autolysosomes. It has been suggested that CHD4, as part of NuRD, regulates at the transcription level molecules related to autophagy through the mTOR pathway (65). Of clinical interest, there is evidence that CHD4 depletion and the presence of trastuzumab also prevents cell proliferation. The authors of the latter study suggest that CHD4 plays a critical role in modulating cell proliferation, the HER2 signaling cascade, and autophagy, allowing speculation that CHD4 could be a target as part of treatment for HER2+ BC.

In hypoxic breast cancer cells, CHD4 physically interacts with hypoxia-inducible factors (HIFs), promoting the progression of BC (**Figure 2D**). In this study, Wang et al. (28), using *in vitro* BC cells (MDA-MB-231 and T47D), described that CHD4 physically interacts with both HIF1 and HIF2 under conditions of hypoxia. CHD4 enhances HIF transcriptional activity independent of its helicase activity and the NuRD complex: it requires mutual binding of CHD4 and HIF to target genes, recruiting RNA Pol II to HIF target genes through p300. Loss-of-function studies of two independent *CHD4*-knockdowns (CHD4-KD), revealed significantly decreased HIF transcriptional activity in hypoxia in MDA MB-231 cells, compared with sh-Scramble. Under hypoxia in these cells, CHD4 enhances the expression of a subset of HIF downstream target genes related to angiogenesis (*e.g.* VEGFA), response to hypoxia, extracellular matrix organization, collagen catabolic processes, apoptosis, and cell proliferation. In addition, functional studies in MDA MB-231 cells have also shown that *CHD4* KD decreases colony growth and cell invasion under conditions of both normoxia and hypoxia. These authors also examined the role of CHD4 in tumor growth in an orthotopic breast cancer xenograft mouse model. Breast tumor growth was significantly attenuated in NSG mice bearing *CHD4*-KD MDA MB-231 cell xenografts. In summary, CHD4 enriched at the chromatin regions near hypoxia response elements (HREs) induces a subset of HIF target genes in breast cancer cells in a setting of hypoxia and xenograft tumor. CHD4 may promote the progression of BC as a coactivator of hypoxia-inducible factors (28).

Further research is needed to determine whether CHD4 is involved in any other pathways that might prevent or promote BC. The *CHD4*-KD studies discussed here highlight the importance of identifying mutations in *CHD4* responsible for CHD4 depletion. This knowledge will help us address how CHD4 mediates the chemotherapeutic response, and thus identify which patients with breast cancer are more likely to benefit from the different treatments. In the absence of studies directly examining the role of *CHD4* mutations in BC, indirect data from endometrial cancer suggest that the hot spot mutations R975H and R1162W disrupt protein function, promoting the loss of *CHD4* in endometrial cancer cells, which induces a cancer stem cell phenotype to promote cancer progression. Hence, these and other loss of function mutations could have the same impact in BC.

### Clinical Relevance of *CHD4* Expression and *CHD4* Mutations in Breast Cancer

The clinical relevance of the role of *CHD4* in BC has been confirmed by the different authors using *in vivo* patient data from various breast cancer databases. Here we assess whether *CHD4* expression level is a marker of poor prognosis, and if depletion of *CHD4* could impact the therapeutical approach used in breast cancer. The clinical data available are summarized in **Table 3** and discussed below.

**Table 3:** Summary of studies describing the clinical relevance of *CHD4* expression as a biomarker of prognosis in different breast cancer patients.

No. patients	Type of breast cancer	*CHD4* expression	Clinical relevanceTherapeutical implications	Reference
208	Breast cancer Oncomine database	mRNA levels & immunostaining	High expression of *CHD4* correlates with low expression of p21. High expression of *CHD4* is a biomarker of poor prognosis. *CHD4* may be a useful target in the treatment of BRCA-proficient BC cells.	(25)
60	TNBC	Immunostaining	Higher *CHD4* expression is positively correlated with metastatic stage, tumor recurrence, and survival status. High *CHD4* expression at the level of mRNA and protein significantly correlated with shorter survival.	(26)
51	TNBC Ualcan and Oncomine database	Immunostaining	Low co-expression of *CHD4* levels and *β1 integrin* correlated with better overall survival. Integrin inhibitors might benefit patients with TNBC and high *CDH4* expression levels.	(27)
382	TNBC	mRNA	High *CHD4* expression positively correlated with HIF target genes and poor overall survival *CHD4* is a powerful candidate in the development of new anti-cancer agents in TNBC.	(28)

*In vivo* data clearly indicate that high *CHD4* expression is a biomarker of poor prognosis in different BC types, especially the more aggressive ones such as TNBC (see **Table 3**). The clinical relevance of CHD4 and its function in p21 regulation in breast cancer have been analyzed using patient tissues and a bioinformatics approach. This study has shown inverse correlation between *CHD4* and p21 expression, suggesting *CHD4* may be a useful target in the treatment of BRCA-proficient BC (25). Some authors showed that overall survival was higher in patients with low co-expression levels of *CHD4* and *β1 integrin* (51 patients, 27). In this latter study, IPA analysis identified 10 proteins involved in CHD4-mediated *β*1 integrin: Snail1 and 2, Notch-1, SMARCA4, JUN, VCAM1, BRD4, CD4, IL4, and MYC. This suggests that some of these proteins could bind to *CHD4* silencing several tumor suppressor genes and regulating oncogenic functions. These findings suggest that *β*1 integrin inhibitors might be of benefit to patients with TNBC who show high *CHD4* expression levels (27).

Wang et al. (28) observed *CHD4* mRNA up-regulation in the BC subtypes luminal A, luminal B, HER2+ and basal-like. Remarkably, *CHD4* up-regulation was mutually exclusive to other known HIF coactivators. Conclusions were that *CHD4* coactivates HIF to promote breast tumor growth, and that different mRNAs were also up-regulated in human breast tumors (ZMYND8, KDM4C (JMJD2C), CDK8, CREBBP (CBP), KAT), suggesting the heterogeneity of epigenetic regulation of HIF in breast cancer. As summarized in **Table 3**, Kaplan–Meier analysis of the TCGA dataset revealed positive correlation between high *CHD4* mRNA levels and the poor overall survival of patients with breast cancer. These authors suggested that *CHD4* is up-regulated and positively associated with HIF target genes in human BC, and that *CHD4* is an independent risk factor for women with BC.

Luo et al. (26), through IHC staining of biopsy specimens from 60 TNBC patients, observed significant correlation between high *CHD4* expression and metastatic stage, tumor recurrence, and survival status in these patients. Kaplan–Meier survival analysis revealed that patients with high *CHD4* expression showed significantly shorter survival compared with patients with low *CHD4* expression. In addition, a multivariate Cox regression model for CHD4 protein expression level and various clinical parameters also identified CHD4 expression as a significant predictor of overall survival. These authors suggest that CHD4 could be a prognostic biomarker in TNBC.

*In vitro* experiments have recently suggested that *CHD4* depletion may be a therapeutic target in different types of BC, as it could lead to reduced tumor proliferation, migration, invasiveness, and growth (27, 28, 61–64) (**Figure 2**). Thus, *CHD4* status could determine the response of BC patients to current treatments, and pharmacological inhibition or targeting of *CHD4* expression could improve the clinical outcome in breast cancer patients according to breast cancer type. Thus, some authors have suggested that *CHD4* depletion (*via* low *CHD4* mRNA expression) might modulate the response to cisplatin in ovarian and BRCA2 breast cancers (63). Pharmacological inhibition of CHD4 may improve treatment in TNBC (61). Further, *CHD4* depletion could increase sensitivity to trastuzumab treatment of HER2+ breast cancer cells (62), and *CHD4* silencing improves sensitivity to cisplatin and PARP1 inhibitor in TNBC cells (25, 26). These data are promising as approximately 15% of BC patients have TNBC, in which neither estrogen/progesterone receptors nor HER2 expression can be detected, and these patients cannot benefit from currently available receptor-targeted systemic therapies. Systemic treatment for patients with triple negative disease is currently limited to chemotherapy, and their survival is poor compared to patients with other cancer subtypes.

For the *CHD4* mutations described here, no sensitivity or resistance data are available for the current pharmacological BC treatments. For the majority of mutations, available data are scarce or inconclusive such that more functional studies are required. We could, nevertheless, speculate that HER2-positive patients carrying R975H (found in HER2+ BC) will be good candidates for HER2-targeted therapy and could have a favorable outcome because of sensitivity to current pharmacological treatments such as trastuzumab. Clinically, the presence of *CHD4* mutations in breast cancer cells creates different epigenetic settings that could impact the progression of breast cancer and modify the response to current therapies. Further, some *CHD4* mutations have been shown to diminish their function, which indicates that interpretation of this information may provide benefit for patients. Compelling evidence indicates that CHD4 is a biomarker of drug sensitivity in cancer cells and that pharmacological inhibition of *CHD4* expression could improve clinical outcome in breast cancer patients.

Some of our results warrant further investigation. Particularly, the frequency and functional and clinical implications of putative driver mutations and those classified as pathogenic need to be investigated in large cohorts. These results, nevertheless, anticipate important implications of *CHD4* for the biological comprehension and prognosis of breast cancer, and point to this gene as a novel therapeutic target for BC patients.

### Summary

The present work shows that somatic mutations in *CHD4* occur at low frequency in BC compared to other gynecological cancers. A total of 81 point mutations in *CHD4* were identified in patients with BC, 19 of which also appeared associated with other cancers. Many of these mutations are located in highly conserved residues of the CHD4 ATPase motor subunit. Some mutations (*e.g.*, R1162W) influence ATPase activity and DNA translocation activity, while others seem to modify protein stability (R877Q/H, R975H) or disrupt DNA binding (R572*, X34_splice). According to data from *in vitro* and *in vivo* studies, some *CHD4* mutations could play a role in breast cancer and confer sensitivity to current pharmacological BC treatments. Low *CHD4* expression may promote either resistance or sensitivity to therapeutic agents [cisplatin, poly-ADP-ribose polymerase (ARP) inhibitor, or trastuzumab] *via* different molecular pathways according to the type of BC in question. However, data on *CHD4* mutations are scarce, and the functional importance of many of the mutations discussed here requires further investigation.

## Data Availability Statement

The datasets presented in this study can be found in online repositories. The names of the repository/repositories and accession number(s) can be found in the article/**Supplementary Material**.

## Author Contributions

PG-A, AN, and AF-S made substantial contributions to conception and design of this study. AN, AR-L, MaG, and MiG acquired and analyzed data. PG-A and AN drafted the article. AF-S, AR-L, MG, FA-S, and MiG critically revised the manuscript for important intellectual content. All authors contributed to the article and approved the submitted version.

## Funding

PG-A is supported by Ministry of Science and Innovation of Spain MICINN (grant no. SAF2016-77816-P). AN, AF-S, MaG, and AR-L are supported by the *Fundación de la Universidad Europea* (project numbers XSAN001907 and XFGU001903). FA-S is a recipient of a fellowship from the MICINN (no. BES-2017-080629).

## Conflict of Interest

The authors declare that the research was conducted in the absence of any commercial or financial relationships that could be construed as a potential conflict of interest.

## Supplementary Material

Supplementary Table 1 Summary of studies and characteristics of *CHD4* mutations in Breast Cancer. Data from cBioPortal, TCGA-BRACA and Cosmic Databases.
